# The Therapeusis of Remittent Fever

**Published:** 1883-09-20

**Authors:** A. M. Pelton

**Affiliations:** Demming’s Bridge, Texas


### THE THERAPEUSIS OF REMITTENT FEVER.

BY A. M. PELTON, M. D., DEMMING'S BRIDGE, TEXAS.

In malarial remittents the indications are: 1. To neutralize the malarial poison. 2. To relieve vascular pressure. 3. To remove morbid secretions. 4. To disperse accumulated heat.

The various preparations of Peruvian bark meet the first indication more nearly than any other; the sulphate of quinia standing at the head of the list. The best antiperiodic effects are obtainable during the remission. The amount of sulphate of quinia given during each remission should be from fifteen to thirty grains in one, two, or three doses, and timed so that the last dose should be given four hours before the beginning of the next exacerbation. If, however, the case is seen but once each day or every second day, it may be a better plan to give eight or ten grains of the sulphate of quinia every eight hours, without much reference to the remissions; or perhaps it may be preferred to give fifteen or twenty grains at each dose, morning and evening, and twelve hours apart.

There are three methods by which to relieve vascular pressure: First, by stimulating the vaso-motor nerves. Second, by diminishing the force of the heart's action. Third, by lessening the amount of fluid in the blood vessels of the congested organs.

The nervous and arterial stimulants, and probably quinine also, have the power of stimulating the vaso-motor nervous system, thus forcing the blood out of the congested internal organs into the general circulation. A favorite prescription with me is Dr. Rezin Thompson's fever syrup, of which the following is the formula :

| | | |
|---|---|---|
| ℞ | Syr. rhei | f. ʒ jv, |
| | Tinct. valerian | f. ℥ ij, |
| | Ol sassafras | gtts. xx, |
| | Piperine | grs. x, |
| | Sup. carb. soda | grs. xx. |

M. Adding the valerian last. Give one tablespoonful every two or three hours in half-glass of water or sweet milk.

Under its use the remissions become more marked and prolonged and frequently at the expiration of twenty-four hours there is a very decided fall of the temperature. In many cases convalescence follows earlier than with any other plan of treatment with which I am acquainted. The following prescription from Dr. Thompson is also very effective in some cases—

| | | |
|---|---|---|
| ℞ | Muc. g. acacia | f ℥ jv, |
| | Morph. sulph | grs. jv, |
| | Ol. terebinth | f ʒ j, |
| | Spts. lavendulus co | f ʒ j. |

M. Give one teaspoonful every two or three hours. Stimulating the vaso-motor nervous system, gives good results in all forms of remittent fever, and especially in the early stage of the sthenic form, and in the adynamic form throughout its course.

Diminishing the force of the heart's action, can be accomplished by the use of nervous and arterial sedatives, such as aconite, gelseminum, veratrum viride, digitalis, chloral, antimony, etc.

The following is an old and effective formula—

| | | |
|---|---|---|
| ℞ | Vin. ant. et pot. tart | *m* xx, |
| | Tinct. hyoscyam | *m* xxv, |
| | Tinct. opii | *m* xv, |
| | Liq. ammon acet | f ℥ j. |

M. Give every four hours.

If chloral hydrate is selected, it should be given in five grain doses every three hours; if veratrum viride, it should be given in doses of one or two drops of the officinal tincture every hour or second hour ; if gelseminum, the dose should be from five to ten minims of the fluid extract every two hours. Where the skin is moist, tincture of digitalis, minims fifteen with a mineral, acid every four hours or six hours, has been highly recommended.

The following is a favorite plan of controlling fever: give a drop or a half drop of the tincture of aconite with one-eighth drop of carbolic acid in a teaspoonful of water every ten or fifteen minutes for one or two hours,, and afterwards hourly or every second hour. Should it produce much prostration with a feeble and weak pulse, then reduce the dose. The carbolic acid is added to relieve any nausea which may be present. Diminishing the force of the heart's action is well adapted to sthenic cases, when there is a reliable and competent nurse, or the physician can give it his personal attention.

The amount of fluid in the congested organs can be lessened by blood-letting, either general or local, by evacuants, by derivatives, or by poultices. Large poultices applied over the abdomen dilate the blood-vessels of the skin, thus taking a quantity of blood out of the general circulation, and they also render the bowels more easily acted upon by laxatives. Stimulating liniments rubbed along the spine and over various portions of the body frequently give great relief; so also mustard sinapisms applied to various parts of the body. Rubbing the surface of the body with tincture of capsicum, or with strong mustard baths in connection with nervous and arterial stimulants are generally very beneficial, and are sometimes followed by profuse perspiration. The method of employing the mustard baths is, to add to tepid water sufficient mustard, then with a cloth apply to a portion of the body with friction, then wipe dry with a coarse towel, and apply to some other part of the body, giving special attention to the back and limbs. These baths should be repeated every six or eight hours.

In most cases the secretions and excretions discharged into the alimentary canal, are lessened in quantity and vitiated in quality. It is very important that the alimentary canal and the glandular aparatus opening into it, should be assisted in their efforts to remove waste and effete products from the system. In fact, quinine and moderate purgation alone are equal to the cure of the majority of malarial remittents. Early in the disease the following powders may be given :

℞ Hydrag, chlor mitis.................... grs. vj to viij,
Soda bicarb.......................... grs. x to xij,
Sacch .............................. grs. xij to xv.

M. Ft. charts No. 3. Give one every two hours.

Should they not act freely on the bowels, follow with either sulphate of magnesia, oleum ricini, or a pill similar to the following ;

℞ Podoyhyllin .............................. grs. ss,
Aloes.................................... grs. viij.

M. Ft. pills No. 4. Give two or three pills for a dose.

The mercurial, sulphate of magnesia, or pills should be repeated as often as is necessary, in order to keep the bowels open. The skin and kidneys will probably receive sufficient medication through the measures already indicated, though it may be desirable to administer some of the salts of potash, or spirits of ætheris nit.

Some practitioners depend on large doses of quinine, from twenty to thirty grains of the sulphate, once, twice or three times daily, to disperse accumulated heat, and also to retard heat production. Others prefer the direct abstraction of heat by the application of cold water, either by sponging, the wet pack, the wet sheet, or cold bath. Frequent sponging of the surface of the body with cold or tepid water, acidulated or not with vinegar, can be made very effective for the dispersion of accumulated heat, and is generally applicable where there is a dry skin. Exposure of the surface of the body to the atmosphere is often beneficial. In private practice the wet pack, the wet sheets, and cold baths are not generally applicable, and so great is the popular prejudice, that the physician who frequently resorts to these methods, will soon be apt to find his practice leaving him; yet excellent results sometimes follow their use.—*Southern Practitioner.*

